# Intraparenchymal Lung Abscess Complicating a Primary COVID-19 Infection in a Patient with Waldenström’s Macroglobulinemia: A Case Report

**DOI:** 10.3390/idr15040039

**Published:** 2023-07-10

**Authors:** Panagiotis F. Mavroudis, Lemonia Velentza, Panagiotis G. Sfyridis, Styliani Papantoniou, Georgios Kranidiotis, Efthymia Giannitsioti, Alexandra Stamati, Dimitrios Schizas, Styliani Gerakari, Emmanouil I. Kapetanakis

**Affiliations:** 1 Second Department of Internal Medicine and Infectious Diseases Unit, Tzaneio General Hospital of Piraeus, 18536 Piraeus, Greece; panos.mavroudis@gmail.com; 2 Department of Emergency Medicine, Tzaneio General Hospital of Piraeus, 18536 Piraeus, Greece; lemvele@gmail.com (L.V.); sgerakari76@gmail.com (S.G.); 3 First Department of General Surgery, Tzaneio General Hospital of Piraeus, 18536 Piraeus, Greece; pgsfyridis@yahoo.gr; 4 First Department of Internal Medicine and Diabetes Center, Tzaneio General Hospital of Piraeus, 18536 Piraeus, Greece; linapapantoniou@gmail.com (S.P.); gekranid@hotmail.com (G.K.); 5 Fourth Department of Internal Medicine, Attikon University Hospital, National and Kapodistrian University of Athens, 12462 Athens, Greece; gianiemi@hotmail.com; 6 Third Department of Internal Medicine, Tzaneio General Hospital of Piraeus, 18536 Piraeus, Greece; asgns95@gmail.com; 7 First Department of Surgery, Laikon General Hospital, National and Kapodistrian University of Athens, 11527 Athens, Greece; schizasad@gmail.com; 8 Department of Thoracic Surgery, Attikon University Hospital, National and Kapodistrian University of Athens, 12462 Athens, Greece

**Keywords:** COVID-19, SARS-CoV-2, pandemic, lung abscess, cavitating lung lesion, Waldenström’s macroglobulinemia

## Abstract

Intraparenchymal lung abscess development associated with severe acute respiratory syndrome coronavirus 2 (SARS-CoV-2) infection is a rare complication, with only half a dozen primary cases having been reported in the literature. We present the case of a patient with Waldenström’s macroglobulinemia who developed a lung abscess subsequent to a primary SARS-CoV-2 infection. We present a 63-year-old male patient with SARS-CoV-2 infection and a history of Waldenström’s macroglobulinemia who developed a cavitating intraparenchymal lung abscess with an air-fluid level in his right lower lobe two weeks following admission to hospital. The patient became septic and developed acute respiratory failure requiring mechanical ventilation and intensive care. He was managed with broad-spectrum antibiotic therapy and aspiration drainage, but unfortunately due to his severe clinical condition died 20 days after his initial admission. The development of a lung abscess in patients with COVID-19, although rare, can be quite compromising and even prove fatal, especially in immunocompromised patients. Clinicians should be aware of this potential complication.

## 1. Introduction

Up until 28 June 2023, 767,518,723 cases of COVID-19 disease had been reported worldwide, with 5,334,160 confirmed cases and 37,174 deaths in Greece [1]. The COVID-19 pandemic started at the end of 2019 in Wuhan, China and, to date, has spread worldwide and has caused over 6.9 million deaths [1]. COVID-19’s primary clinical characteristics include myalgia, fever, cough, respiratory distress and dyspnea [2]. The causative agent of this pandemic is a ribonucleic acid (RNA) coronavirus called severe acute respiratory syndrome coronavirus 2 (SARS-CoV-2), causing various pathological conditions in humans, mainly including respiratory failure and acute respiratory distress syndrome (ARDS) as well as heart failure, renal failure, liver damage, thrombosis, shock and multi-organ failure [2]. Especially in the respiratory system, SARS-CoV-2 causes either primary or secondary pneumonia as a result of bacterial infection [3]. Treatments and other interventions used for COVID-19 are mostly supportive since no specific drugs have been found to completely treat COVID-19 [2]. However, several drugs have been repurposed to check their efficacy against COVID-19 and more than 600 clinical trials are currently ongoing [2].

The incidence of lung abscess as a complication of primary SARS-CoV-2 infection is low, with just 25 reported cases, of which only 7 (average age = 49.3 years, six males/one female), have been reported in non-intubated patients [3,4,5,6,7]. A lung abscess presents as a cavity of the pulmonary parenchyma usually filled with fluid and necrotic debris and is caused by bacterial infection and lung tissue necrosis [4,6,8]. It is associated with significant morbidity and mortality, despite antibiotic treatment, with a frequency of 15–20% [6]. Several factors predispose someone to the development of lung abscesses, including immunosuppression, poor oral hygiene causing dental abscess, intravenous drug abuse and alcoholism, whilst their etiology can be polymicrobial [4,6,8].

Herewith, we present a case of a lung abscess which developed in a patient with Waldenström’s macroglobulinemia and SARS-CoV-2 infection.

## 2. Case Presentation

A sixty-three-year-old male unvaccinated patient diagnosed with SARS-CoV-2 pneumonia was admitted via the emergency room and hospitalized in one of our hospital’s COVID-19 isolation wards. His presenting symptoms included protracted fever of up to 38 °C, chest pain and mild hypoxia. The patient’s history included Waldenström’s macroglobulinemia diagnosed 3 years prior, for which he was receiving long-term ibrutinib therapy, which was well-tolerated with no reported side effects and which produced good disease control. The patient reported no recent travel abroad and had no history of allergy, tobacco, any pulmonary disease, drug or alcohol use, loss of consciousness or poor oral hygiene. There was no family history of lung disease or respiratory-failure-related death reported. In his initial physical examination, he had a normal blood pressure and pulse, he was febrile with a temperature of 38.6 °C, his respiratory rate was slightly labored with 24 breaths/minute and his SpO_2_ was 93% with a PO_2_ of 71 mm Hg on room air. Lung auscultation revealed crackles mainly at the right lower base, with the rest of the physical examination being unremarkable. Initial diagnosis of SARS-CoV-2 infection was performed via an antigen test which was confirmed with a nasopharyngeal swab polymerase chain reaction (PCR) test. This, although not confirmed by genomic sequencing, which is not routinely performed, most likely represented infection by the beta variant which, at the time and according to nationwide epidemiological studies, represented the predominant COVID-19 strain in Greece. 

**Figure 1:**
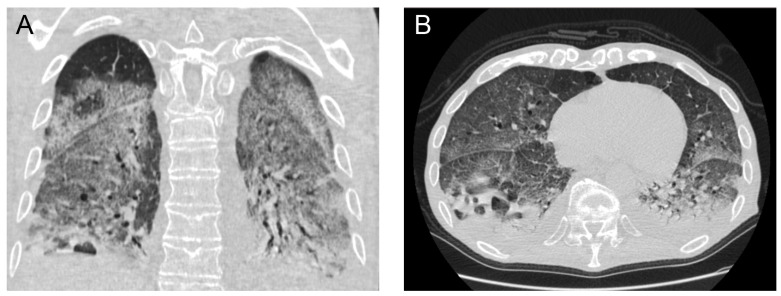
Initial (on admission) chest computed tomography scan in coronal (**A**) and axial (**B**) views demonstrating extensive bilateral areas of consolidation and ground glass infiltrates, with lung cavitation mainly on the posterior segment of the right lower lobe.

Laboratory investigations showed normal lymphocyte count with neutrophilia (89%) and severe lymphopenia (4.9%), increased C reactive protein (CRP) at 10.3 mg/L and normal procalcitonin (PCT) at 0.82 µg/dL. The rest of his laboratory values were within the normal range. His chest X-ray on admission presented with extensive consolidation and infiltrates mainly in the lower lung fields with blunting of the costo-diaphragmatic angles. His admission chest computed tomography (CT) scan demonstrated extensive ground glass infiltrates and diffuse consolidation bilaterally with a focus on the right lower lobe (Figure 1). 

The patient was initiated on the therapeutic regimen established at the time for COVID-19 infection, which included intravenous (IV) ceftriaxone 2 g once a day, a remdesivir loading dose of 200 mg followed by 100 mg once a day for 10 days, dexamethasone 6mg once a day per os and enoxaparin thromboprophylaxis 40 mg once a day subcutaneously. He was also treated with high-flow oxygen therapy and his hypoxia and general condition gradually improved to the point that he was able to mobilize independently and was almost weaned off supplemental oxygen. 

However, on the 12th day of hospitalization he suddenly and acutely developed signs of sepsis with marked leukocytosis (White Cell Count = 19,020/µL), elevation in his inflammatory markers (CRP = 24.0 mg/dL, PCT = 2.8 µg/L), hypotension, lethargy and acidosis. His respiratory function became rapidly compromised, once more requiring increased levels of oxygen therapy and, ultimately, he had to be intubated and mechanically ventilated due to type 1 respiratory insufficiency. The patient was consequently transferred to the intensive care unit (ICU).

**Figure 2:**
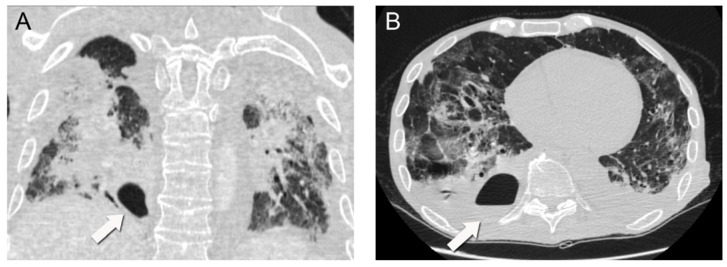
Subsequent chest CT scan demonstrating a 7 × 4 cm^2^ intraparenchymal abscess (arrows) with an air-fluid level in the posterior basal segment of the right lower lung lobe (coronal (**A**), axial (**B**)).

A repeat chest CT scan demonstrated multiple converging consolidated lesions in both lungs with scattered areas of ground glass. Most notably, in the posterior basal segment of his right lower lung lobe, an intraparenchymal thin-walled cavity measuring 7 × 4 cm^2^ in diameter was observed. The presence of an air-fluid level was indicative of a pulmonary abscess associated with the previous areas of dense consolidation (Figure 2). It was hypothesized that the pulmonary abscess was possibly associated with a bacterial superinfection caused by the COVID-19 disease. Due to unstable respiratory conditions, bronchoscopy was not performed. However, direct cultures were obtained via thoracentesis under ultrasound guidance and fluid aspiration. Both blood and aspirated fluid cultures failed to identify a causative microorganism. 

Conversely, initial empirical antibiotic therapy with ceftaroline fosamil 600 mg twice a day IV to cover community cocci was initiated but, following an infectious disease consultation and because the patient’s condition continued to decline, antimicrobial treatment was modified as follows: meropenem 2 g three times a day IV, colistimethate 50,000 U/kg/day IV and linezolid 600 mg twice a day IV. A thoracic surgery consultation was also obtained, but it was deemed that the patient was not a candidate for surgical resection due to his poor clinical status and increased associated risk.

Despite intensive therapeutic management and support, his condition deteriorated quickly and significantly and the patient passed away 20 days post initial hospital admission and 2 days post ICU admission.

## 3. Discussion

Inter-parenchymal abscess development appears to be a rare complication of SARS-CoV-2 infection and is mostly associated with bacterial superinfection [2,4,8]. The majority (90%) of superinfections are polymicrobial in nature with causative organisms including both aerobic (*Staphylococcus aureus*/Methicillin Resistant Staph. Aureus (MRSA), *Streptococcus pyogenes*, *Klebsiella pneumonia*, *Pseudomonas aeruginosa*, *Hemophilus influenza*, *Acinetobacter*, *Escherichia coli* and *Legionella*) and also anaerobic (*Bacteroides fragilis*, *Fusobacterium capsulatum* and *Peptostreptococcus*) species [8]. Although information in the literature is limited, the causative organisms in lung abscess development in patients with COVID-19 appear to be the same [3].

Lung abscess treatment remains a challenging process and depends on several factors, such as the size and location of the abscess, response to antibiotic regime, as well as the clinical status of the patient [8]. Pulmonary abscesses are usually treated conservatively, and this approach is also supported for patients with SARS-CoV-2 infection by the limited published evidence available [3,4,5,6,7]. 

Therefore, the optimal therapeutic management in patients with a lung abscess includes a combination of antibiotic therapy depending on causative organism(s) and diagnostic thoracentesis under ultrasound or CT guidance to obtain fluid from the air-fluid cavity which can then be cultured in order to isolate the causative organism(s) of the secondary infection [8,9]. Duration of antibiotic therapy varies, but usually ranges from 5 to 21 days of intravenous antibiotics followed by 28–48 days of oral application [8]. Serial chest X-rays need to be performed to monitor the size and appearance of the cavity. Surgical treatment is indicated when the abscess has a diameter of more than 6 cm, if the patient remains symptomatic for more than 12 weeks with appropriate antibiotic therapy or if the patient is severely septic [8]. Surgical options include open tube thoracostomy or per cutaneous trans thoracic tube drainage via local anesthesia or surgical resection of the lung abscess along with the surrounding tissue [8]. 

Chest tube drainage is a relatively easy-to-perform surgical procedure which is indicated in about 11–21% of patients after failure of antibiotic therapy [8]. Its efficacy when utilized approaches 84% with a complication rare of 16% and a mortality incidence of 4% [8]. Complications of drainage include spillage of necrotic detritus with infection of the pleural cavity and formation of a pyopneumothorax, empyema or bronchopleural fistula [8,9]. Per cutaneous trans thoracic tube drainage via a Seldinger technique under ultrasound or CT guidance is preferred over open thoracostomy as it is safer and better tolerated by patients [8,9].

Surgical resection as definitive treatment of lung abscess is warranted in approximately 10% of patients [8]. Indications can be acute, such as hemoptysis, prolonged sepsis, pyopneumothorax and empyema, or chronic, such as unresponsiveness to antibiotic therapy of more than 6 weeks, bronchopleural fistula and diameter over 6 cm [8]. Lobectomy, if possible, is the resection of choice, but atypical (wedge) resections or segmentectomies can also provide satisfactory results if the abscess and surrounding necrotic tissue are adequately removed [8]. In certain cases, the authors have performed unroofing and debridement of the cavity with good results and this is something also supported by the literature [8]. 

In this patient, because of his quite unstable condition due to septic shock, a conservative treatment approach was pursued. A thoracotomy and surgical excision was deemed too high risk considering the marginal clinical condition of the patient. Instead, an ultrasound-guided aspiration was performed with the removal of 140 cc of purulent fluid; unfortunately, no organisms grew from the culture of this fluid. 

Empirical antibiotic therapy with ceftaroline fosamil was initiated on the assumption of a community-acquired cocci (both *Steptococcus* and *Staphylococcus*) infection. These seem to be the predominant causative microorganisms for lung abscess formation in adults [8]. Effective response to antibiotic therapy can be seen in 3 to 4 days following the initiation of antibiotic therapy and the patient’s general condition will usually improve after 4–7 days [6]. Unfortunately, because of his immunocompromised status and the weakened clinical condition caused by the primary SARS-CoV-2 infection, the superinfection and the associated lung abscess were quite detrimental, causing his death.

The development of cavitating lesions with an air-fluid level following viral pneumonia have been previously described [7]. They are the result of a necrotizing process in areas with initial consolidation and superinfection [7]. 

The patient’s initial chest CT demonstrated significant areas of diffuse consolidation and patchy ground glass opacification. These are findings which have previously been associated and described with COVID-19 viral pneumonia [7]. In addition, in hindsight there seemed to be a focus of atelectasis and consolidation in the right lower lobe which could represent the precursor of the lung abscess.

The patient’s Waldenström’s macroglobulinemia and the long-term treatment with ibrutinib he was receiving made him immunocompromised and, thus, more prone to a bacterial superinfection and subsequent development of a cavitating lesion and abscess. Ibrutinib is a Bruton tyrosine kinase (BTK) inhibitor which blocks the B-cell receptor pathway, which is often aberrantly activated in Waldenström’s macroglobulinemia, producing an uncontrolled clonal proliferation of terminally differentiated B lymphocytes [10]. Recently published guidelines do not recommend the cessation of BTK inhibitor therapy in Waldenström’s macroglobulinemia patients with asymptomatic or mildly symptomatic COVID-19 [10]. On the contrary, cessation of treatment has a risk of causing an IgM rebound and associated constitutional symptoms (which can be confused as COVID-19-related) [10]. In addition, in severely symptomatic COVID-19 patients, ibrutinib’s immunosuppressive effect, although it can initially decrease the response to COVID-19, may be of benefit in reducing late and severe immune-mediated complications [10]. There is thus some scant evidence that ibtutinib therapy may act protectively against pulmonary injury in COVID-19 infection, producing a lower rate of pulmonary complications [11]. Consequently, considering the above and also the lymphocyte profile of the patient, ibtutinib therapy was maintained during the initial admission period and was only discontinued upon the onset of severe sepsis and respiratory impairment. 

## 4. Conclusions

This case adds to the limited literature on the infectious complications of COVID-19 pneumonia, which clinicians should be aware of and vigilant for. The development of a lung abscess in COVID-19 patients, especially if they are immunosuppressed, although rare, can be quite compromising and even prove fatal. 

## Data Availability

Not applicable.
